# Recommendations to support the mental health and wellbeing of response-focused civil servants asked to work from home during public health emergencies in the United Kingdom: a Delphi study

**DOI:** 10.1186/s12888-025-07604-7

**Published:** 2025-11-28

**Authors:** Charlotte E. Hall, Samantha K. Brooks, Neil Greenberg, Dale Weston

**Affiliations:** 1 Department of Psychological Medicine, Weston Education Centre, King’s College London, London, SE5 9RJ UK; 2 Behavioural Science and Insights Unit, Science, Strategy and Evidence, Chief Scientific Officer Group, UKHSA, Porton Down, Salisbury, SP4 0JG UK; 3 Health Protection Research Unit, Institute of Psychology, Psychiatry and Neuroscience, King’s College London, 10 Cutcombe Road, London, SE5 9RJ UK

**Keywords:** Occupational health, Civil servants, Mental health, Resilience, Workforce resilience

## Abstract

**Background:**

A recent research project sought to explore the experience of UK response-focused civil servants asked to work from home during the COVID-19 pandemic and to create a series of recommendations for future public health emergencies requiring response-focused homeworking. In general, civil servants from one select government organisation were able to convey a plethora of lessons learnt, reflections on experience, and suggestions for future support offers should another public health emergency require homeworking. Recommendations were derived from the evidence, but it remained important to ensure that the recommendations were useful, fit for purpose, and actionable. This required collaboration with experts in appropriate fields (in this case, mental health, occupational health, and government affiliated individuals).

**Method:**

This Delphi study sought to refine and operationalise evidence-based recommendations designed to support the mental health and wellbeing of future UK response-focused civil servants asked to work from home during public health emergencies. A total of 32 experts were recruited into the study; these individuals provided feedback and rated recommendations over three iterative rounds.

**Results:**

A total of 19 recommendations reached consensus at a > 90% level of agreement. In line with feedback from experts, these were categorised into preparedness and response focused recommendations to aid with implementation. Collectively, these recommendations establish the importance of organisation/employer level initiatives to protect mental health and wellbeing in response-focused civil servants working from home.

**Conclusion:**

The key outcome of this work has been the importance of employees, managers, and organisations retaining flexibility when working from home, as a range of factors (e.g., living circumstances, others in household, relationships with colleagues) can influence experience, and each employee will face theird own barriers and facilitators to working from home.

**Supplementary Information:**

The online version contains supplementary material available at 10.1186/s12888-025-07604-7.

## Introduction

In January of 2020, the World Health Organization (WHO) declared “a public health emergency of international concern” over a novel coronavirus outbreak (later named as COVID-19). Following a period of rapid transmission of the virus, a global pandemic was declared on the 11th of March 2020 [1]. The UK Government put in place several behavioural interventions to reduce transmission of the COVID-19 virus. These methods forced the public to restrict their contact with others and highly impacted usual day-to-day life. For example, the Prime Minster announced that people would be only able to leave their homes in certain circumstances, namely, to shop for necessities as infrequently as possible, exercise (a maximum of one activity per day), and to gain medical care for oneself or care for vulnerable others [2]. As of the 16th of March 2020, the public were instructed to “start working from home where possible” [3].

Nearly half of the UK Public in employment reported to work from home during April of 2020 [4] in comparison to 5% prior to the pandemic [5]. This therefore raises the question as to what the likely consequences of working from home are, for both employees and employers. Pre-pandemic, homeworking was often seen by employees as a way of overcoming difficulties (e.g. decreasing or eliminating commuting time [6]) and was therefore viewed as being advantageous. However, homeworking sometimes also had negative connotations, and resulted in challenges for employees. For example, experiencing blurred boundaries between work and home life, feelings of constant connectivity to the workplace [7]. Line managers also face challenges in the working from home context. For example, being unable to see employees results in difficulty checking in with individuals [8], as well as difficulties ensuring employees are remaining motivated and working productively whilst at home [9]. Furthermore, creating communication channels and creating strong, trustworthy, and honest working relationships with direct reports may be more difficult over a computer screen [10]. Organisations, or employers, also face struggles with employees working from home. For example, ensuring staff abide by organisational policies (e.g., in relation to break taking), are remaining productive and undistracted, and are working safely and comfortably [11].

The COVID-19 pandemic brought upon an unprecedented situation of prolonged and enforced working from home for a vast majority of the UK workforce. The change to the way of working occurred suddenly, and as a result, many of the preparatory steps recommended for effective remote working (e.g., ensuring safe, comfortable and appropriate remote workplaces and technical equipment) were not carried out in time [12]. In addition, there were a vast number of additional household challenges brought upon by the COVID-19 pandemic, homes were now both workplaces and learning environments (for those with children in the household) and many in employment were furloughed so were spending more time at home [13]. The impact of working from home during the pandemic has been mapped in terms of mental health, productivity, and wellbeing; consistently mixed findings are apparent, with many reports establishing a negative or equivocal impact at best [14–17].

While the negative experiences of various occupations working on the frontline during the COVID-19 pandemic are well documented amongst current literature (e.g., healthcare workers [18, 19], teachers [20, 21], mental health professionals [22]), one occupation with seemingly limited literature surrounding their experience of working from home (whilst being classified as a frontline responder) are UK response-focused civil servants. These are civil servants who were contributing to, and providing, effective delivery of the coronavirus response for local or national government; [23]. A recent project seeking to explore the experiences of this occupational group was carried out by the authors of this article, with the aim of developing recommendations to protect the mental health and wellbeing of future responding civil servants asked to work from home during public health emergencies. In total, two reviews of literature (one systematic [17] and one review of reviews [24]), a cross-sectional survey and secondary data analysis [25], and a series of interviews [11] were conducted as part of this research project. In brief, this programme of research has established that civil servants reported feeling a strong sense of purpose and achievement for contributing to the COVID-19 pandemic response, but in turn, expressed the workload and demands were very high – particularly for those who had to rapidly, and unexpectedly, transition from office or lab work to home working (less so for those who joined the Civil Service during the pandemic). Many expressed that they were underprepared to work from home and were able to convey a plethora of lessons learnt, reflections on experience, and suggestions for future support offers should another public health emergency require homeworking. Reflecting on experiences during the COVID-19 pandemic provide vital lessons for working during, and responding to, future public health emergencies.

Indeed, at each stage, where applicable, evidence-based recommendations seeking to protect, maintain, and even improve the mental health and wellbeing of UK response-focused civil servants during public health emergencies were derived from the findings by the research team. However, to ensure that the recommendations are appropriate, actionable, and achievable, it was important to collaborate and consider the opinions of experts in appropriate fields (in this case, mental health, occupational health, and government affiliated individuals).

Therefore, using an expert panel, the current study sought to gain consensus on, as well as iterate, and operationalise a series of evidence-backed recommendations seeking to protect and maintain the mental health and wellbeing of UK response-focused civil servants during public health emergencies.

## Method

A Delphi study is a widely used method to obtain input from a group of experts [26]. It is a process commonly used in health science focused research [27], and provides the opportunity to seek, expert opinion in a structured manner. Delphi studies are usually carried out over a number of rounds to allow for reflection (i.e., each round is built on previous findings and allows for responses to be reconsidered) [26]. Experts also remain anonymous to one another throughout the study, as anonymity allows for experts to express views freely, without judgement, and without bias (e.g., group conformity; [28]).

### Experts

A total of 32 experts were recruited into the Delphi study. A purposeful sampling technique using professional networks was carried out to ensure representation across occupations and areas of expertise. We sought to include a range of team or department leads in the organisation, alongside experienced response-focused employees who had working from home whilst responding to public health emergencies (i.e., experts by experience). As a result, experts were selected due to knowledge and expertise in mental health and wellbeing (*n* = 10), occupational health or occupational psychiatry (*n* = 5), behavioural science (*n* = 1), human resources (*n* = 1), or were affiliated with the UK Government (*n* = 15).

### Design

In brief, three iterative stages with the experts took place between March and June of 2024 (in line with other Delphi study research, e.g [29]). The method used allowed engagement with a 32 stakeholders, in a structured manner, to generate and prioritise recommendations to support and maintain the mental health of home-based UK response focused civil servants during public health emergencies. Online surveys were used to collect expert opinion and feedback. All communication with Delphi study members was carried out via email. A Delphi reporting checklist was used [30] and can be found in Supplemental information 1.

#### Round 1

To begin, the experts were asked to review and feedback on a series of recommendations derived from previous research stages (supplemental information 2). No rating was conducted in this round. Instead, the focus was on improvement and development of the recommendations.

Experts were presented with 38 recommendations via an online survey hosted on Qualtrics, each recommendation was accompanied by a free-text response box to collect feedback. Experts were asked to comment on individual recommendations and to identify whether any should be adjusted/modified, whether any recommendations or concepts were missing and should be added, or if any recommendation should be removed entirely. Additional space was also provided at the end of the survey to collect any general feedback (e.g., for any additional recommendations, any key concepts missing from the recommendations as they stood).

The data was de-identified by the first author for the purpose of analysis and research team discussions. The original recommendations were edited where required, with any additional recommendation suggestions analysed using content analysis to form new recommendations to be rated in the next round. Where conflicting feedback was received, two members of the research team (CEH, SKB) independently made changes to the recommendations and outcomes (if conflicting) were discussed. All new recommendations were discussed by the research team to ensure they aligned with evidence derived from the project and could be supported by the conducted research.

All recommendations then progressed to round 2 (i.e., those remaining the same between rounds 1 and 2, those which had been edited/amended, and any new additional recommendations).

#### Round 2

The second round consisted of experts ranking each of the recommendations via an online survey hosted on Qualtrics. Participants were asked to rate the importance of each recommendation on a five-point scale (1 = should not be included; 2 = not important; 3 = Do not know/ depends; 4 = important; 5 = essential) based on other Delphi study research (e.g [29, 31]. An open-ended question also allowed participants to submit additional comments or recommendations to be included in the subsequent and final round of the survey.

Once all experts had responded, the results were analysed to establish consensus on the importance of each recommendation. An initial consensus level of 80% was applied [26] (i.e., 80% of experts must deem the recommendation ‘important’ or ‘essential’). But following analysis it was decided to increase the consensus level to >90% due to high levels of initial agreement (i.e., 64% reaching >80% agreement at the first rating).

Each recommendation was then sorted into one of the following three categories:

1. Recommend and retain (i.e., > 90% of experts must have deemed the recommendation ‘important’ or ‘essential’).
2. Re-rate (i.e., > 70% but < 90% of experts must have deemed the recommendation ‘important’ or ‘essential’).
3. Reject (i.e., < 70% of experts deemed the recommendation ‘important’ or ‘essential’).

Recommendations classed as re-rate progressed into Round 3. Amendments were made to recommendations following expert feedback, and these were re-entered into Round 3. Lastly, any new recommendations derived from expert feedback were also entered into Round 3.

#### Round 3

The third round consisted of experts ranking each of the recommendations via an online survey hosted on Qualtrics. In Round 3, experts were invited to rate all recommendations carried over from Round 2, in the same format as described previously.

When being sent the link to the survey, each expert that completed Round 2 was sent a personalized report. This report summarised the findings of Round 2 and provided a list of items that had been recommended and rejected by the panel. For re-rate items, experts were also sent their previous rating of each recommendation in comparison to the group as a whole.

Following analysis, each recommendation was then sorted into one of the following two categories:

1. Recommend and retain (i.e., > 90% of experts must have deemed the recommendation ‘important’ or ‘essential’).
2. Reject (i.e., < 90% of experts deemed the recommendation ‘important’ or ‘essential’).

### Ethical considerations

This study did not require ethical approval, in line with King’s College London’s ‘*Is my project Research*,* Service Evaluation or Audit?*’ tool. This was also corroborated by the Health Research Authority ‘*is my project research?*’ Decision tool [32].

The British Psychological Society Code of Ethics and Conduct [33] was followed nonetheless. All experts consented to take part and were able to withdraw at any point during the Delphi study. It was decided to keep the expert panel anonymous from one another, as well as to the research team where possible, to allow for all comments and opinions to be considered fairly and without bias. When feedback was collected, the lead author removed personal data (in this case, name of expert) before analysis, and research team members only saw blind copies of the data. Please note, at the start of the study experts were asked if they would like to be acknowledged on any reports or outputs coming from this study – to which 70% agreed. This was checked again when the study was completed, with an additional two members wishing to be acknowledged on any outputs. The remaining experts remain anonymous.

## Results

### Round 1

Round 1 was conducted between the 25th of March and 12th of April 2024 and was used to generate feedback on 38 evidence-backed recommendations (Supplemental information 2). Specifically, experts were asked to feedback on what they thought was missing from the recommendations, what needs amending or improving, and which recommendations they believed could be combined or removed entirely. A total of 31 experts completed the Round 1 survey.

**Fig. 1:**
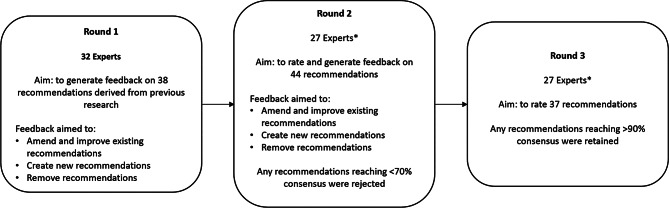
Flow diagram of the Delphi study over three iterative rounds. Note: *the same 27 experts were consistent across Rounds 2 and 3

Of the initial recommendations, six received supportive feedback and advanced to the next round without amendment, and ten were removed as they were deemed unimportant or were deemed by experts to be covered by other recommendations. The remaining 22 recommendations were modified and expanded, where appropriate, resulting in 30 recommendations going into Round 2 (Supplemental information 2). Additionally, a further eight recommendations were derived from expert feedback, and were added for review in Round 2. Figure 1 displays a flow diagram of recommendations added/rated/rejected in each round).

Feedback from experts indicated overlapping constructs and concepts so recommendations were grouped into “preparedness”, “managers”, “provisions”, “support and guidance”, “social connections”, and “organisational culture” for presentation to experts across the next two rounds for ease and clarity.

### Round 2

Round 2 was conducted between the 2nd of May and 21st of May 2024 via online survey and was used to gain feedback on the 44 recommendations from Round 1 and for experts to rate how important they believe each recommendation is to protect the mental health and wellbeing of response focused UK civil servants. A total of 27 experts completed the Round 2 survey.

Following analysis, 11 recommendations were accepted (> 90% consensus), five were rejected (< 70% consensus) and the remaining 28 fell into the re-rate threshold (> 70% but < 90%). Due to expert feedback, 12 of the re-rate recommendations were adjusted and 11 new recommendations from expert feedback were added into Round 3. Resulting in 37 recommendations being assessed in Round 3 (Supplemental information 2).

### Round 3

Round 3 was conducted between the 30th of May and 20th of June 2024 via online survey and was used for experts to rate how important they believe each recommendation is to protect the mental health and wellbeing of response focused UK civil servants. A total of 27 experts (the same experts that completed Round 2) completed the Round 3 survey.

Following analysis, eight recommendations were accepted (> 90% consensus). As there was no fourth round, the remaining recommendations were rejected (< 90% consensus). In summary, by the end of Round 3, a total of 19 recommendations gained expert consensus at a 90% agreement level (which can be found in Table 1), with two recommendations reaching 100% consensus.

**Table 1:** Recommendations reaching > 90% expert consensus of importance

Recommendation	Percentage agreement
Organisations should have a plan or strategy in place for communicating with employees in public health emergencies.	100
Organisations should seek to provide employees with adequate resources and guidance about how to maintain one’s mental health and psychological resilience when working from home. It is noted that this should ideally be readily available, as an element of organisational preparedness.	100
Managers should aim to be contactable and approachable to their direct reports but remain comfortable in setting clear boundaries and protecting their time when necessary.	96
Organisations asking employees to work from home during public health emergencies should seek to quickly provide equipment and training to allow employees to, as best possible, work safely and comfortably from home.	96
Organisations should provide guidance on what support and provisions are available to employees needing to build workspaces at home (e.g., what is offered, how need is assessed, relevant non-pay benefits).	96
Organisations should seek to foster an environment where employees support one another and communicate well (e.g., checking in on one another, problem solving).	96
Organisations should ensure IT support for employees should be easily accessible, as well as seek to be fast, responsive, and available across all hours of working.	96
Organisations should make sure that employees are aware and able to access their offer of support resource(s), including signposting to appropriate services outside the organisation.	96
Organisations should seek to provide high-quality, evidence-based wellbeing support accompanied by actively demonstrating care towards employees and provide practical support where needed.	96
Organisations should aim to have a virtual induction process for new employees and include an information pack about the organisation (e.g., aims, mission statement, working arrangement, response context and purpose, key contacts).	93
Organisations should seek to do what they can to avoid reactive approaches to the way of working changing and should instead focus on ensuring that they are proactive and prepared for dealing with the way staff may have to work during emergencies.	93
Managers should be open to negotiate with employees in relation to working flexibility (i.e., if core working hours are adhered to, and business needs met, employees should be able to have flexibility on how they work as some colleagues have preferences of early starts, late finish, compressed hours).	93
Due to individual differences and contexts apparent whilst working from home, managers should seek to understand and support their employees on a case-by-case basis (i.e., by taking into account any additional factors that may impact their work.	93
Managers should be proactive in providing resources and opportunity to bolster team cohesion, resilience, working relationships, and good communication practices (e.g., through facilitating informal chats, group activities, away days).	93
In longer term emergencies (e.g., pandemic response), where isolation may be apparent, organisations should allow and implement time for employee directive initiatives (e.g., weekly team debriefs, social events).	93
Organisations should provide employees the opportunity to feedback and review working practices.	93
Organisations should consider alternative arrangements for employees without access to a suitable workspace (e.g., due to space restraints, caring responsibilities).	92
There should be a general focus on maintaining workers mental health and wellbeing when they are asked to work from home for a prolonged period, and mitigating any concerns about poor mental health	92
Organisations should recognise and trust that despite not being visible in the workplace, employees continue to contribute to organisational objectives while working at home efficiently.	92

All recommendations not reaching consensus by Round 3 can be found in Supplemental information 2. Additionally, an example of recommendation development across three rounds can be found in Supplemental information 3.

### Final outcomes

**Fig. 2:**
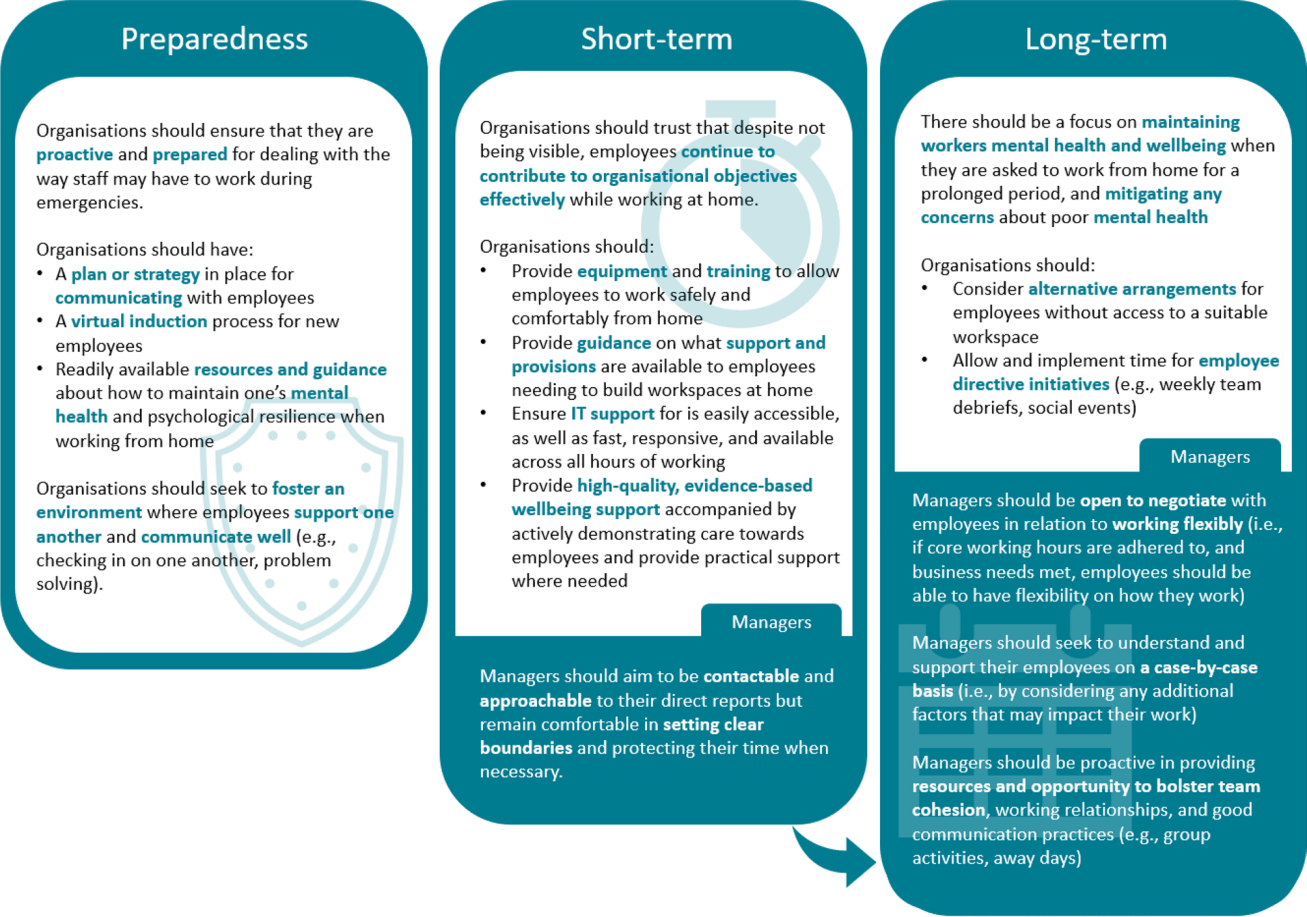
Expert-approved, evidence-backed recommendations to support the mental health and wellbeing of UK response-focused civil servants asked to work from home during public health emergencies

A total of 19 recommendations reached consensus of importance. Expert feedback consistently noted that that many of the proposed recommendations were more, or less, applicable depending on the context in which they were being applied particularly in relation to short vs. long-term working from home. In the Round 3 survey (after all recommendations had been rated), experts were thanked for their time and asked, ‘*to provide any thoughts/feedback on which recommendations or concepts are most applicable to short-term or long-term stints of working from home*’ to aid with the implications of this research. Based on the feedback received, the recommendations were grouped into ‘preparedness’, ‘short-term’ or ‘long-term’. A visual representation of this is presented in Fig. 2 which incorporates all recommendations in a condensed format for ease. In line with feedback, short term-recommendations are more logistically focused (e.g., equipment, IT support, wellbeing guidance) and relate to getting employees being able to work from home well. In contrast, longer-term recommendations are more focused on improving employee experience, such as considering potential alternatives (e.g., flexible working, workspaces) and allowing time for inclusive and accessible employee led initiatives to aid team connections and reduce isolation.

## Discussion

The current research sought to gain expert consensus on evidence-based recommendations seeking to support the mental health and wellbeing of UK response-focused civil servants asked to work from home during public health emergencies. Additionally, expert feedback was used to tailor the evidence-based recommendations into appropriate, actionable, and achievable recommendations to be within the population of interest. To do this, a panel of 32 experts in mental health and occupational health, alongside experienced civil servants were recruited into a three-round Delphi study. A total of 19 recommendations reached expert consensus at a 90% agreement level, with two reaching 100% consensus. The vast majority of the recommendations agreed upon by experts related to organisations or employers (*n* = 15), followed by recommendations for management (*n* = 4). Only minimal recommendations referenced actions employees themselves can take or contribute to, and these also required organisational focus (*n* = 2).

Looking across the final recommendations detailed in the preceding section, it is apparent that most are tailored towards organisations or employers rather than individuals. The importance of implementing organisational level initiatives to provide support for employees is not unique to this project. For example, a Delphi study also seeking to recommend best practice to protect the mental well-being of midwives and nurses in the UK also concluded with many recommendations focused on what employers can do for their employees [29]. Indeed, these findings align with the outcomes of the current study that suggest protecting employee mental health is the duty of employers, as the vast majority of expert-approved recommendations were tailored towards employer level recommendations.

Preparedness recommendations included both recommendations which reached 100% consensus by experts, establishing the importance of organisational preparedness. The sudden change to ways of working during the COVID-19 pandemic highlighted a lack of organisational preparedness; this is consistent with previous literature emphasising that, for example, the rapid nature of the transition meant that many key organisational policies could not be carried out (i.e., ensuring employees have the correct equipment to work safely and comfortably; [12]). As recommended in the current paper, organisations should seek to be proactive and prepared, and therefore have plans and strategies in place for communicating with employees and inducting new employees, as when employees are asked to work from home, normal methods of communication are changed and conversing with employees (e.g., to share updates and information), becomes more difficult. For example, if email distribution lists are chosen as the main method of communication, these should be frequently updated to reflect employee turnover to ensure all relevant employees are reached.

Secondly, when considering working as a frontline employee from your own home (i.e., in the case of response-focused civil servants) providing an effective and productive response may be more difficult due to a range of personal and contextual factors, or barriers, to overcome [24]. For example, having inappropriate workspace available, a lack of relevant training, or having competing demands (such as household chores, caring responsibilities) [24]. Experts in the current study agreed that organisations should seek to provide employees with adequate resources and guidance about how to maintain one’s mental health and psychological resilience when working from home, which may provide employees with the resources and strategies to overcome these new potential challenges. Additionally, it is suggested for organisations to foster an environment where employees support one another and communicate well, which may be addressed by providing more time for employees and teams to work collaboratively to aid building social capital which can provide an additional network of support during public health emergencies [34].

In relation to short-term working from home for response-focused civil servants during public health emergencies, experts prioritised recommendations that were centred around getting employees to be able to work from their homes comfortably, and safely, in a timely fashion. Responding to a public health emergency results in increased work demands (e.g., quick turnaround deadlines, workload [11]), it is therefore important to ensure that, in the short term, employees have all of the necessary resources to start working from home productively. For example, recommendations reaching consensus promoted the importance of: provision of equipment and appropriate training to be able to work from home; guidance and support on what provisions (and potential non-pay benefits) are available to employees seeking to build workspaces at home; ensuring that IT support is easily accessible and responsive across all working hours; and the provision of high-quality, evidence-based wellbeing support for employees where required.

Longer term recommendations focused on overcoming issues and maximising experiences that may be more relevant for long-term homeworking. For example, as working from home becomes longer term, separation from colleagues can result in feelings of isolation (e.g [11, 24]), and logistical concerns (e.g., lack of workspace, or particular equipment) can become more apparent [35]. Recommendations reaching consensus suggested the importance of: considering alternative workspaces for those without appropriate space at home (e.g., potential other places of working, or office hire); and, allowing more opportunities for social activities/meets to overcome the potential loneliness and isolation that may be apparent with long-term working from home arrangements.

Furthermore, across the range of recommendations, the role of the manager in protecting and maintaining employee wellbeing is documented as important. Research suggests that a good line manager - employee relationship can positively impact employee experiences [25, 36–38]. Specifically, experts reached consensus on four recommendations relating to manager actions, one of which was applicable to short term, and three applicable to longer term emergencies. In the short term, experts highlighted that managers are also employees, and they too need to be able to protect their mental health and wellbeing when responding to a public health emergency. Therefore, managers are recommended to be contactable and approachable to their employees but remain comfortable in setting clear boundaries and protecting their time when necessary. In longer-term recommendations, the role of the manager becomes more prominent and a resource for employees to draw from, for example in terms of maximising benefits (e.g., flexibility in line with workplace demands in terms of role, timings, location (as much as possible)) and overcoming difficulties of working from home (e.g., implementing social activities/meets to overcome loneliness and isolation).

### Limitations

The current study sought to establish a list of evidence-informed and expert-backed recommendations for the future of working from home during public health emergencies for response-focused civil servants. Despite achieving this aim, there are limitations to consider. Firstly, a very high level of agreement was taken (90%), and despite this resulting in more rigorous and relevant outcomes, it means that many useful recommendations may have been excluded from the study. This is a pitfall of the Delphi method, as experts may disagree with others, which means that recommendations experts may personally feel would be useful are ultimately excluded as not enough other experts agree on importance [29, 39]. However, all recommendations within this study are evidence-based and are all are included within supplementary information so that interested practitioners or researchers can access and use them. Secondly, for the current study, it was decided to keep the experts anonymous from one another in order to examine contributions equally. This however meant that open discussion found in some Delphi studies (e.g., using workshops as in [40]) was not carried out. However, this approach is in line with a well-established method recommended to ensure all contributions are appraised equally within Delphi studies [28]; in addition, the high level of agreement provides further confidence that the identified recommendations did not require further discussion. Lastly, there was some attrition between rounds: the study began with 32 experts and only 27 experts contributed to Rounds 2 and 3. However, in comparison to attrition rates in other Delphi studies (e.g., expectations of a 20% over three rounds [41]) the current study shows good levels of retention (i.e., 32 experts to 27 over three rounds; 15.63% attrition rate).

## Conclusion

This Delphi study sought to reduce, refine, and improve derived recommendations seeking to support the mental health and wellbeing of future UK response-focused civil servants asked to work from home during public health emergencies. A total of 32 experts were recruited into the study, 27 of whom provided feedback and rated recommendations over all three iterative rounds. A total of 19 recommendations reached consensus at a > 90% level of agreement, and in line with feedback from experts – these were categorised into preparedness, short-term, and long-term recommendations to aid with implementation. We recommend that organisations and managers maintain a good degree of flexibility in term of how they support their employees; rather than a one size fits all, as this study recommends that a case-by-case approach to supporting mental health and wellbeing is employed where possible. From an organisational perspective, this can potentially be done by providing employees with a plethora of optional resources, guidance, and training opportunities, along with appropriate signposting to non-organisational resources, about how to maintain one’s mental health and psychological resilience when working from home, allowing employees to seek and use the resources as and when required.

## Supplementary Information

Below is the link to the electronic supplementary material.

Supplementary Material 1

Supplementary Material 2

Supplementary Material 3

## Data Availability

All recommendations rated by experts can be found in the supplementary information. Experts did not provide consent for their feedback on the recommendations to be publicly available.
