# Supplementary material for: Recommendations to support the mental health and wellbeing of response-focused civil servants asked to work from home during public health emergencies in the United Kingdom: a Delphi study
Source: BMC Psychiatry. 2025 Nov 28;25:1203. doi: 10.1186/s12888-025-07604-7 (PMC12751646; doi:10.1186/s12888-025-07604-7)

**Example of recommendation development**

Recommendations were routinely developed in line with expert feedback, and each and every recommendation used in the current study across all three Delphi rounds can be found in the additional supplemental information files. This supplementary information provides a worked example to provide an example of the process.

The initial evidence-based recommendation, as presented in Figure S1, was: “*Employees should be able to implement social activities, meeting or catch ups to allow them to connect with other members of staff (e.g., chair yoga, online games)”*.

In Round 1, expert feedback mainly related to how employees should not be carrying out social activities in working hours without organisation/employer approval. There was a general appreciation and understanding of the importance of social activities under certain contexts (e.g., pandemic response), but the term ‘*online games*’ was not liked by experts. Additional operationalisation was also incorporated into the recommendation (i.e., *seeking to create and maintain social connections with colleagues and improve wellbeing vs. allow them to connect with other members of staff).* Therefore, the recommendation was iterated to: “*Organisations should promote time for, and encourage, employee-directed initiatives (e.g., team catch-ups, chair yoga) seeking to create and maintain social connections with colleagues and improve wellbeing)”*.

In Round 2, the recommendation was initially rated as unimportant by experts (reaching <70% agreement). Additional feedback was provided by experts, meaning that the recommendation could be adjusted and re-considered in Round 3 (30, 31). The key feedback was centred around the social aspect of working from home only being applicable in longer term emergencies (e.g., pandemic response), where isolation and loneliness is more likely to impact employee wellbeing. Additionally, examples of potential social activities were also changed, to be more team focused and open to interpretation (e.g., *social events* vs *chair yoga*). Resulting in the recommendation being iterated to: “*in longer term emergencies (e.g., pandemic response), where isolation may be apparent, organisations should allow, and implement time for, employee directive initiatives (e.g., weekly team debriefs, social events)*”.

In Round 3, this recommendation was accepted at a >90% expert consensus level. Thus, demonstrating the value of integrating expert feedback into the recommendations (i.e., <70 to >90 agreement).

**Figure 1:** Example of how recommendations were developed across rounds with expert feedback.


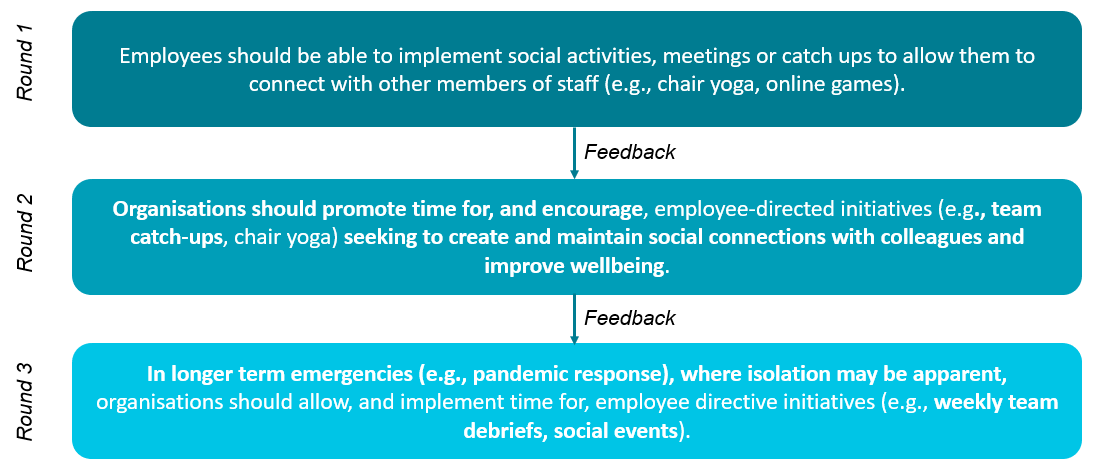

Supplement: Supplementary file 3 — Supplementary Material 3 [file 12888_2025_7604_MOESM3_ESM.docx]
